# cGAS-STING pathway in cancer biotherapy

**DOI:** 10.1186/s12943-020-01247-w

**Published:** 2020-09-04

**Authors:** Yang Wang, Jingwen Luo, Aqu Alu, Xuejiao Han, Yuquan Wei, Xiawei Wei

**Affiliations:** Laboratory of Aging Research and Cancer Drug Target, State Key Laboratory of Biotherapy, National Clinical Research Center for Geriatrics, West China Hospital, Sichuan University, No. 17, Block 3, Southern Renmin Road, Chengdu, Sichuan 610041 PR China

**Keywords:** cGAS-STING pathway, Cancer biotherapy, Interferon, Cyclic dinucleotide, Agonist, Delivery system, Clinical trials

## Abstract

The activation of the cGAS-STING pathway has tremendous potential to improve anti-tumor immunity by generating type I interferons. In recent decades, we have witnessed that producing dsDNA upon various stimuli is an initiative factor, triggering the cGAS-SING pathway for a defensive host. The understanding of both intracellular cascade reaction and the changes of molecular components gains insight into type I IFNs and adaptive immunity. Based on the immunological study, the STING-cGAS pathway is coupled to cancer biotherapy. The most challenging problem is the limited therapeutic effect. Therefore, people view 5, 6-dimethylxanthenone-4-acetic acid, cyclic dinucleotides and various derivative as cGAS-STING pathway agonists. Even so, these agonists have flaws in decreasing biotherapeutic efficacy. Subsequently, we exploited agonist delivery systems (nanocarriers, microparticles and hydrogels). The article will discuss the activation of the cGAS-STING pathway and underlying mechanisms, with an introduction of cGAS-STING agonists, related clinical trials and agonist delivery systems.

## Introduction

Cancer biotherapy replies on stimulating the body’s anti-tumor biological response by initiating the host’s defensive mechanisms and using biological agents. By killing cancer cells and inhibiting its growth, cancer treatment is to regulate the balance between immune responses and tumors to treat cancer, whose mechanism is different from that of chemotherapy, radiotherapy and surgery [1, 2]. Current cancer biotherapy includes cancer vaccine [3], cancer immunotherapy [4], cancer gene therapy [5] and anti-angiogenesis therapy [6]. In the early years, the therapeutic effect was witnessed in tumors with strong immunogenicity, especially melanoma [7]. But now, cancer biotherapy research has been spread into breast cancer [8], lung cancer [9] and liver cancer [10]. Meanwhile, anti-neovascularization drugs, monoclonal antibodies, cancer vaccines, gene therapy drugs and immunomodulators have been used clinically and been studied in clinical trials.

Tumor immune surveillance is illustrated as the immune system’s recognition and the elimination of malignancies. Once tumor-associated antigens are perceived, innate immunity, adaptive immunity and cytokines function together to fight against tumors [11]. In long-term coexistence of the immune system and cancer cells, tumors alter tumor-associated antigens to directly escape immune surveillance. Moreover, tumors remodel their mesenchymal surroundings to support their growth, which is called tumor microenvironment [12]. Under the increasing level of tumor variation, selectively immune pressure and imbalance between immunity and tumors, tumors escape from the surveillance by immunoediting [13]. Innate immunity plays an important role in recognition of exogenous nucleic acids, especially cytoplasmic DNA sensing by the cGAS-STING pathway. Normally, foreign DNA is degraded by nucleases, such as three prime repair exonuclease 1 (TREX1) and ribonuclease H2 (RNase H2) [14, 15]. The destruction of cellular homeostasis would cause cytoplasmic DNA accumulation, such as endogenous retrovirus, DNA damage, genomic instability, damaged mitochondria, dying cells, exosomes, DNA viruses, retroviruses and bacteria [16–18]. Under such circumstances, cyclic guanosine monophosphate-adenosine monophosphate synthase (cGAS) senses and is activated when binding to double-stranded DNA (dsDNA). In the energy-consuming process, cGAS transforms adenosine 5′-triphosphate (ATP) and guanosine 5′-triphosphate (GTP) into cyclic GMP–AMP (cGAMP). As a secondary messager, cGAMP with other cyclic dinucleotides (CDNs) transmits the signal to the downstream endoplasmic reticulum (ER) protein named stimulator of interferon genes (STING). Then, the signal cascades and culminates in interferon regulatory factor 3 (IRF3) and NF-κB targets in the nucleus, leading to the secretion of type-I interferon (IFN). IFN is important to tumor-specific T cells [19, 20].

cGAS-STING pathway challenges the traditional pathogen-specific structural patterns as an innate immunity’s defensive system. Therefore, to maintain the immune balance, the pathway requires regulatory mechanisms from different levels to guarantee correct immune responses. In the review, we will discuss the knowledge of how DNA is sensed by cGAS-STING pathway, describing why cellular homeostasis happens, what the signal is initiated by, how molecules co-ordinates with each other to execute the command and how the signal is protected from erroneous immune activation. We will also focus on the molecular mechanisms and biological effects of an activated cGAS-STING pathway to enhance cancer biotherapy efficacy. Furthermore, we will review cGAS-STING agonist delivery systems and related drugs.

## The molecular mechanisms of cGAS-STING pathway

### cGAS activation by pathogens and dsDNAs

As stimuli, endogeneous retrovirus, DNA damage, genomic instability, damaged mitochondria, dying cells, exosomes, DNA viruses, retroviruses and bacteria contribute to the production of dsDNA. Herpes simplex virus-1 (HSV-1), vesicular stomatitis virus (VSV) and porcine circovirus type 2 (PCV2), as DNA viruses, promote IFN by activating cGAS-STING signaling pathway [21–23]. Besides, endogeneous retrovirus RNAs triggers a cascade wave of signaling to upregulate INF production by cGAS-STING pathway [24]. For instance, human immunodeficiency virus type 1 (HIV-1) contains single-strand DNAs of stem-loop structures, which activate cGAS in a sequence-dependent way. Mitochondrial DNA is released through outer membrane pores by activating BAX/BAK, followed by the detection of cGAS [25]. Not only in human somatic cells, but also in bacteria, cGAMP signaling activates the phospholipase which destroys bacterial membrane integrity to death before phage reproduction. It provides an evolutionary point to microorganisms against phages [26].

Structurally, how the interaction of dsDNA and cGAS activates cGAS has been studied. As an important functional domain of cGAS, C-terminal nucleotidyltransferase (NTase) domain consists of a catalytic domain and two positive parts. Promoted by zinc ribbon, dsDNA activates cGAS by forming 2:2 cGAS-dsDNA complexes. Each cGAS contains a dsDNA like a ladder network to stabilize the cGAS-dsDNA structure. Then, the stable structure turns on the switch of rearrangement of the catalytic domain to transform GTP and ATP into cyclized cGAMP. The synthesized cGAMP containing two phosphate diester bonds show a higher affinity for STING. Although other nucleic acids (ssDNA, ssRNA and dsRNA) also bind to cGAS, they cannot rearrange the catalytic domain [26, 27]. Another important N-terminal domain is responsible for maintaining the liquid phase of dsDNA and cGAS. By forming droplets of them together, it is facilitated for dimerization and subsequent activation, especially with the help of zinc ions [27, 28]. Notably, such liquid phase is sensitive to the concentration of cGAS and dsDNA. Only when there exists a certain level of dsDNA, cGAS is activated.

### Intracellular Cascade reaction of cGAS-STING signal transduction

Activated cGAS produces cGAMP and further binds to STING for activation on the ER membrane. Before STING activation, dimerized STING shapes as V containing two C-terminal domains on the ER surface. Once activated, STING alters into a more closed conformation and transfers to Golgi by ER-Golgi intermediate compartment (ERGIC), which initiates downstream signal cascades [28]. On reaching Golgi, palmitoylation of two cysteine residues is essential for the location of STING on Golgi and allows for both STING oligomerization and TANK Binding Kinase 1 (TBK1) activation [28]. Together with the motif pLxIS in STING, TBK1 is allowed to co-activate IRF3 by phosphorylation. The phosphorylated IRF3 polymerizes and translocates to the nucleus to regulation downstream transcriptional factors of which interferon is the most representative hallmark [29, 30].

### cGAS-STING-mediated molecular changes

**Fig. 1:**
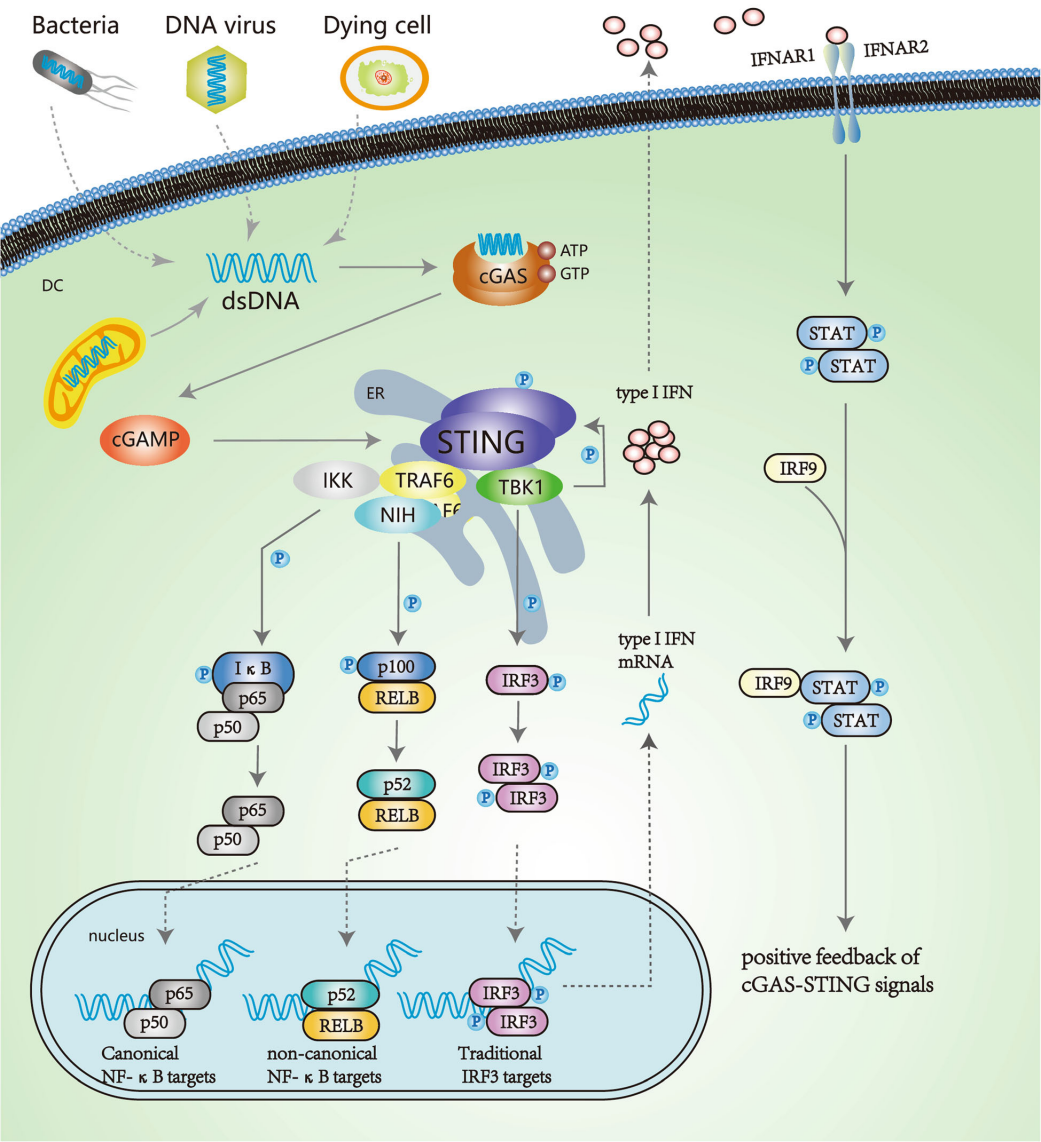
the cGAS–STING pathway

The activated cGAS-STING signal pathway contributes to the increase of IRF3, non-canonical NF-κB and canonical NK-κB, respectively [31]. TBK1 and STING co-phosphate IRF3 (interferon regulatory factor 3). Dimerized IRF3 imports to the nucleus to target corresponding genes. Similarly, like TBK1, mitogen-activated protein kinase kinase kinase 14 (MAP 3 K14/NIK) and IκB kinase (IKK) are other kinases recruited by activated STING. NIK phosphorylates nuclear factor kappa B subunit 2 (NFKB2/p100) combined with RELB. RELB is a proto-oncogene comprising one of the NF-kB subunit. After phosphorylated p100 is degraded by the proteasome to p52, p52 and REBL form into a heterodimer to elicit non-canonical NF-κB signals [32, 33]. Interestingly, the production of canonical NF-κB signals resembles that of non-canonical one. Kinase IKK phosphorylates NFKB inhibitor alpha (NFKBIA/IκB) for the recognition of proteasomal degradation. Thus, heterodimer p65/p50 is separated from IκB/p65/p50 complex to the nucleus, eliciting canonical NF-κB signals [34]. Dimer IRF3, heterodimer p52/RELB and p65/p50 all serve as transcriptional factors. IRF3 regulates the expression of IFNB1 in the nucleus. IFNB1 translation in the cytoplasm results in producing type I IFN, secreting out of cells. Type I IFN stimulates tyrosine kinase-associated receptor, IFNAR1/IFNAR2 heterodimers, which phosphorylates STAT1/STAT2. The process seems like the JAK-STAT signal pathway [35]. The difference is that IRF9 joins phosphorylated dimer STAT/STAT. IRF9/ STAT/STAT as a transcriptional factor transactivates cGAS, developing positive feedback of cGAS-STING signals (shown as Fig. 1) [36].

cGAS-STING signals trigger inflammation and inhibition of cell proliferation and apoptosis. Activation of the cGAS-STING pathway lead to inflammation by rising the concentration of inflammatory cytokines in innate immune responses. We would witness fibrotic changes in chronic diseases [37]. Immunogenicity is enhanced in lymphocytes and other antigen presenting cells (APCs) by high cGAS-STING pathway activity. The mechanism is the activation of NF-κB activation and STING which both activate those immune cells [38]. Sensitive T lymphocytes’ proliferative characteristics are hindered in activated STING cells due to active NF-κB signals [39]. During adaptive immune responses, cGAS-STING signals induce apoptosis for active STING. The phenomenon is often observed in cancerous T cells related to IRF3 and other proapoptotic genes, like p53 [40, 41].

## Activated cGAS-STING pathway and cancer biotherapy

### Mechanisms of IFNs as immune sensor initiating adaptive immune response

Activated cGAS-STING pathway renders the massive production of type I IFNs. As a ligand, IFN binds to two subunits of the IFN receptor. Human type I IFN is a family of multiple cytokines, containing 13 IFNα subtypes, IFNβ, IFNγ, IFNκ and IFNε [42]. Accumulated evidence shows that T cell infiltration is an indication of the production of type I IFNs. Patients with advanced melanoma, who received oncolytic virotherapy, have increased CD8 ^+^ T cells and elevated IFN-γ production [21]. Consistently, clinical data indicate that high levels of extravasating T cells induced by IFN-γ are related to improved survival in patients with colorectal cancer. Those patients with a sound immune system could benefit from biotherapy [43]. On the contrary, tumors are able to abolish IFN formation and reduce T cell recruitment with a lectin secreted by tumors [44]. Therefore, understanding underlying mechanisms among innate immune responses, adaptive immune responses and the cGAS-STING pathway is crucial. Type I IFNs attract cytotoxic T cells to tumor bed [45] and trigger type I T helper cell (Th1) responses [22]. Moreover, type I IFNs make dendritic cells (DCs) mature and present-tumor specific antigens to CD4^+^ and protective CD8^+^ T cells [46]. T cell-inflamed tumors contain type I IFN signature. CD8^+^ T cells show efficient innate priming in triple-negative breast cancer [47]. Overall, these results indicate that type I IFN production is associated with T cell responses against tumor antigens. Mechanically, deficiency in the Ca^2+^ sensor stromal interaction molecule 1 (STIM1) activates STING spontaneously with subsequently increased type I interferons in immunodeficient mice [48]. In vitro, the expression of Ifnb1, Il6 and interferon-stimulated genes (ISGs) were apparently increased in Stim1^−/−^ murine embryonic fibroblasts (MEFs) compared with those in wild-type (WT) cells. Like murine cells, STIM1-deficient patients exhibited increased type I IFN, pro-inflammatory cytokines and upregulated ISGs. Type I IFN promotes DCs antigen cross-priming in certain mechanisms. It reduces the rate of endosomal-lysosomal acidification to maintain cell-associated antigens in RAB5+ and RAB11+ compartments [30]. Furthermore, IFNα promotes antigen presentation ability of DCs by re-locating MHCI molecules to antigen storage compartments in DCs [29]. In addition, Type I IFN upregulates molecules on the DC surface, such as MHCI, MHCII, CD40, CD80, CD86 [31, 32]. These cluster of differentiation molecules are responsible for providing membrane-associated co-stimulatory signals. Collectively, the results suggest that the innate immune system recognizes cancer cells, leading to the activation of certain signaling pathways and the production of type I IFN, which is crucial for antigen cross-priming.

### cGAS-STING signaling pathway initiates type I IFN production and adaptive immunity

Given that IFNs are necessary for the anti-tumor effect, the question that the binding of receptors and ligands causes IFNs production and immune cells’ anti-tumor effect should be understood. Cytosolic DNA activates cGAS further synthesizing cyclic dinucleotides which activates STING. STING gathers around the nucleus and fosters the activation of TBK1. TBK1 phosphorylates IRF3 which transfers signals to downstream type I IFNs gene transcription. Moreover, mutation of STING has been identified in human beings and mice. A heterozygous gain-of-function mutation of STING causes familial chilblain lupus, indicating increased type I IFNs disorders [49]. Patients with STING N154S mutation have STING-associated vasculopathy with onset in infancy (SAVI) whose pathological and pathophysiology changes are pulmonary fibrosis and autoinflammatory disorders. Similarly, STING N153S knock-in mice are vulnerable to develop pulmonary fibrosis after virus infection due to increased type I IFN production [50]. Mechanistic studies found that a low dose of STING agonist ADU-S100 (S100) compatible with checkpoint inhibitors (CPIs) activated tumor-specific CD8 ^+^ T cells and enhanced more intensive anti-tumor immunity. TNFα is dispensable for the management of tumor regression [51]. Immunologically, the production of type I IFNs is engaged in the priming of T cells against tumor-associated antigens (TAAs), intratumoral accumulation of dendritic cells and cross-priming of tumor antigen-specific T cells [52–54]. Therefore, the cGAS-STING signaling pathway is an important innate defensive sensor that monitors the presence of tumors to mature DCs and promotes T cell priming against TAAs.

The function of the cGAS-STING signaling pathway is identified in several mouse tumor models. In Brca-deficient ovarian cancer models treated with olaparib, the number of intratumoral CD4^+^ and CD8^+^ T cells increased, which was accompanied by increased IFNγ and TNFα secreted by themselves. DCs capable of presenting antigens were found to be recruited in the tumor environment (TME). The phenomenon occurred due to the activation of the cGAS-STING pathway and the detection of tumor-derived DNA [55]. Shannon et al. found that mice with ovarian cancer treated with cisplatin had high levels of type I IFN production and inflammation, representing the stimulation of the cGAS-STING pathway. The activated pathway upregulated immunity-recognitive markers. Besides, other non-ovarian solid tumor also showed therapeutic efficacy after intra-tumoral cGAMP administration. Unexpectedly, the cGAS-STING signaling pathway and inflammation have pro-tumor functions [56]. In this study, intra-tumoral injection with STING agonists appeared anti-tumor effect exhibited by T cell responses and regressive tumors in multiple mice tumor models. Meanwhile, experiments invitro suggested that STING agonists administration generated IFNβ production by APCs through the cGAS-STING signaling pathway. STING agonists also contributed to other cytokines production and DCs maturation. Given that 5, 6-dimethylxanthenone-4-acetic acid (DMXAA) capable of disrupting blood vessels induced TNFα by stromal cells in vivo and that its therapeutic efficacy attenuated in TNFR^−/−^ mice, the mechanism of STING agonists therapeutic effect may result from TNFα-mediated angiolysis [57]. In syngeneic ovarian cancer (ID8) and colon cancer (CT26) mice models, poly (ADP-ribose) polymerase inhibitors (PARPi) promoted the levels of phosphorylated Irf3 and STING, which implies active cGAS-STING signaling pathway in vivo. In these PARPi-treated tumor mice models, increased percentages of CD8+ T cells and PD-L1+ cells were observed. These results suggested that PARPi treatment activates the cGAS-STING signaling pathway and rises the levels of type I IFNs and tumor infiltrating lymphocytes (TILs) to trigger an immunogenic response [58].

### STING-cGAS signaling pathway applied to Cancer biotherapy

Now that STING-cGAS signaling pathway plays an important role in sensing tumors in innate immunity, two aspects are deserved paying attention to before we develop effective and safe drugs. First, whether tumors could afford STING-cGAS pathway activation by generating T-cell-rich TME should be taken into consideration. After STING adjuvant administration, TME with T cells is related to the activation of the STING-cGAS pathway, indicating that cancer patients react to such biotherapy and show improved prognostic conditions. The failure of generating activated cGAS-STING signals implies a dysfunctional reaction. Therefore, key mlecules in the pathway may serve as predictive biomarkers to direct cancer therapy. Specifically, BATF3-lineage DCs play a central role in anti-tumor immunity [59]. Before pIRF3 translocating to the nucleus, phosphorylation of TBK1 and IRF3 is essential to transfer signals. So BATF3-lineage DCs and pIRF3/pIRF3 are indicators of allowing researches to proceed. Second, therapeutic strategies should conduct clinical trials to prove their bio-therapeutic potentials. Taken one of STING adjuvants for example, 5, 6-dimethylxanthenone-4-acetic acid (DMXAA) is a flavonoid compound used as vascular disrupting agents [60]. Its immunomodulatory function has been proven to have anti-tumor activity in mice [61]. Clinically, DMXAA’s pharmacokinetics showed well-defined efficacy in cancer patients rolled in phase I clinical trial [62]. When DMXAA was combined with carboplatin to conduct a phase II study in untreated patients with advanced non-small cell lung cancer (NSCLC), the therapeutic efficacy of DMXAA was reflected in several parameters, such as tumor response rate, median survival and median time to tumor progression [63, 64]. However, in a randomized phase III placebo-controlled trial, after received DMXAA and platinum-based therapy, patients with advanced NSCLC showed no difference in overall survival (OS), progression-free survival (PFS) and adverse events [65]. Surprisingly, DMXAA only augments murine cGAS-STING signals, instead of that signals in humans [65], which may account for the dysfunction of treating cancer patients in clinical trials. Therefore, DMXAA has exerted concerns about species specificity when people are developing new drugs. Two years later, a newly-designed C7-functionalized DMXAA derivative was synthesized and further evaluated. It shows an affinity to human STING [66]. Together, developing new STING agonists requires human STING to be interacted and activated before clinical trials.

Apart from the stimulus of cGAS-STING agonist, locally increased level of type I IFNs in TME is another challenge to enhance innate immunity. There are two methods: the safety delivery system and targeted radiation. First, developing a rapid and effective delivery system is imperative to increase drug concentrations in the peripheral and central tumor sites. Tumor-targeted mono-antibody (mAb) conjugated with tumor-inhibitory IFNα can induce cancer cell apoptosis. The key technique in the conjugate is Dock-and-Lock method (DNL) to produce 20-2b. 20-2b is an immunocytokine comprising four cytokine groups. Every cytokine group bonds to one anti-CD20 mAb, suggesting that the conjugate has supreme efficacy [67, 68]. IFNAR-deficient tumors are unable to be sensitive to chemotherapy if type I IFN is artificially recharged [69]. Hence, secreting type I IFN in the TME is an essential process for immune priming. Another important point is whether the significant toxicity of delivery systems would produce, suggesting that we need to further observe the effective half-life and dose of drugs in the TME. Nanoparticles (NPs) of chitosan/poly γ-glutamic acid impair the invasion of colorectal cancer cells due to their immunostimulatory characteristics [70]. Castro et al. used NPs carrying IFN-γ to evaluate the therapeutic effect. These IFN-γ-NPs up-regulated costimulatory molecules on cell surfaces and promote to secrete pro-inflammatory cytokines. The mechanism behind T cell proliferation and inhibitory effect against colorectal cancer cells is upregulated CD40 and CD86 molecules [71]. Stephan et al. tested that injecting tumor-reactive T cells do not have a therapeutic effect on pancreatic ductal adenocarcinoma. Subsequently, they used an implantable biopolymer carrier that loaded with CAR T cells and STING agonists to inhibit solid tumors. Consequently, the complex causes the activation of antigen presenting cells, initiation of lymphocyte responses, shrinking of tumors and elimination of metastases [72]. However, the study of these combined agents needs safety evaluation before clinical development.

Targeted radiation is a second method to activate innate immune responses. Irradiated tumors suffer from DNA damage, modulation of signal transduction and changes of TME, exerting anti-tumor responses. Current studies demonstrated that type I IFN-dependent anti-tumor effect after radiation is mediated by the cGAS-STING pathway, rather than the TLR pathway. Administration of cGAMP can reinforce the intensity of the cGAS-STING pathway, consolidating adaptive anti-tumor ability [73]. Hence, radiation is a key initiator for immune priming in the cGAS-STING pathway. The possible mechanism is that radiation-mediated cancer cell death causes tumor-derived DNA to be released. DCs in the TME sense the alteration and subsequent T cell priming coordinates with anti-tumor immune responses. However, the dose of radiotherapy needs to be identified. Exposure to a high dose of ionizing radiation can lead to adverse effects on patients or animals. Low dose may produce inadequate biological responses [74, 75].

### Cancer vaccine and cGAS-STING pathway

Cancer vaccines are used to induce immune responses to fight against cancer by stimulating the immune system. The first cancer vaccine is reported in 1983 that Mr. William B. Coley inoculated live bacteria into Soft tissue tumors. He found that some tumors shrunk after inflammation. Researching and developing new cancer vaccines is popular nowadays. In 2006, the FDA approved the first cervical cancer vaccine to lower the risks of HPV16 and HPV18 infection and to prevent cervical cancer. To date, most of the cancer vaccine is under clinic trials. Those trials involve cancer patients who are not bearable during chemotherapy, radiotherapy and operation. Thanks to different antigen constituents, cancer vaccines include cell-based vaccine, virus-based vaccine, DNA-based vaccine, protein- and peptide-based vaccine, anti-idiotype vaccine and carbohydrate-based vaccine. Human tumor antigens have low immunogenicity so that adjuvant is added to support cancer vaccine. Then, cytotoxic T cells are activated and humoral immunity is induced. Current researchers have found correlations between the cGAS-STING pathway and those cancer vaccines. In the part, we would introduce the cGAS-STING pathway agonists.

#### DMXAA

DMXAA is a flavonoid compound used as tumor vascular disrupting agents (Tumor-VDAs), which is also named as ASA404 and Vadimezan. Tumor-VDAs have two classes of the family: tubulin-binding class and flavonoid class. The flavonoid class is characterized as the induction of inflammatory responses by the innate immune system in the TME. DMXAA is productive to combine with radiation, hyperthermia and chemotherapy reagents. A good illustration of such a combination is DMXAA with carboplatin and paclitaxel in untreated advanced NSCLC. Co-administration was well-tolerated. There is no cardiac adverse events or ophthalmic abnormalities [63]. DMXAA is a STING agonist. A surprising event is that human STING cannot be induced by DMXAA, compared to the fact that mice STING potentiates innate immune responses by DMXAA. Scientists are continuously searching for DMXAA function in the human body and seeking solutions in those disappointing phase III trials [65]. Mechanistically, DMXAA stimulatory function of NF-κB is also detected in monocytes [76], endothelial cells [77] and tumor cells [78]. Increased NF-κB signals give rise to the production of inflammatory cytokines, affecting various immune cells in the TME. The possible mechanism is that DMXAA not only promotes phosphorylated IRF dimers to translocate to nucleus, but also co-stimulates canonical NF-κB targets and non-canonical NF-κB targets simultaneously.

#### CDNs

CDNs are another kind of cGAS-STING pathway agonist. In the late 1980s, the discovery of them has been found in bacteria as secondary signals. In the pathway, upon interaction with cytosolic DNA, cGAS would synthesize cyclic di-nucleotides to bind to STING for phosphorylation, thus activating STING. Apart from prokaryotic cells, CDNs are thought to participate in innate immune responses of mammalian cells [79]. CDN is a collection of cyclic dinucleotide family, consisting of cyclic di-GMP (c-di-GMP), cyclic di-AMP (c-di-AMP), cyclic AMP-GMP (cGAMP). Amongst themselves, cyclic AMP-GMP contains 3′3’-cGAMP, 2′3’-cGAMP, 3′5’-cGAMP and 2′5’-cGAMP. 2′5’-cGAMP is found to potentiate the activation of human STING due to 2′-5′ linkage [80]. These CDN agonists have anti-cancer potentials. For example, TriVax is a multiple peptide mixture, consisting of CD8 T cell epitope, polyinosinic-polycytidylic acid adjuvant and costimulatory anti-CD40 antibodies. TriVax alone and the combination with c-di-GMP are injected into mice with subcutaneous B16 melanoma cells respectively. C-di-GMP is identified to strengthen TriVax’s function. Co-administration of c-di-GMP slows down tumor growth more intensively. In vitro, adding c-di-GMP to TriVax favors T cells to recognize the B16 tumor cells [81]. Another important case in point is that the anti-tumor efficacy of c-di-GMP is dose- and frequency-dependent. Mice with metastatic breast cancer administrated low dose c-di-GMP are observed elimination of metastases. Meanwhile, 4 T1 tumor cells receiving high dose c-di-GMP are killed. The underlying mechanism is that low dose c-di-GMP promotes myeloid-derived suppressor cells (MDSC) to produce IL-12 for the improvement of T cell responses. High dose c-di-GMP activates caspase-3 for apoptosis. Based on the fact above, low dose c-di-GMP for several times, accompanied with high dose c-di-GMP administration for once, shows no efficacy difference, compared with co-administration of tumor-associated antigen (Mage-b) and c-di-GMP. The explanation is the cross-presentation of TAA by corresponding T cells [82]. Together, enhancing the cGAS-STING pathway by STING adjuvant c-di-GMP is a promising way for cancer biotherapy.

cGAMP is *a co*mmon CDN molecule, stimulating the immune system and exerting tumor clearance [83, 84]. Notably, cGAMP enhances the cGAS-STING pathway not only by binding to STING, but also by rendering neighboring cells stimuli via transport vesicles and intercellular gap junctions [85, 86]. Furthermore, cGAS-STING signals have been identified in different kinds of tumors, such as B16-BL melanoma, MC38 colon carcinoma and RMA-S lymphoma [87]. In multiple mice models, like 4 T1 breast cancer, squamous cell carcinomas, CT26 colon cancer and B16F10 melanoma, injecting cGAMP generates accumulated macrophages. During the process, those macrophages secrete TNFα and chemokines recruiting T cells, indicating that cGAMP-induced anti-tumor effect is involved with macrophages [83]. Inhalation of nanoparticles with cGAMP in mice with lung matastases is another treatment, which distributes cGAMP in lungs rapidly and promotes APC to produce type I IFNs. Additional fractionated radiation can further strengthen the anti-tumor ability and prolong mice survival [88]. In this study, intra-tumoral injection of cGAMP potentiates CD8^+^ T cell responses in melanoma and colon cancer mice models respectively. Based on STING agonists promoting immune responses, co-injection of immune checkpoint inhibitors (anti-CTLA-4 and anti-PD-1) further enhances anti-tumor effect [89, 90]. Together, cGAMP, as cGAS-STING pathway agonist, has versatile applications in anti-tumor immunity.

#### Other agonists

Except for classic CDNs, STING additionally includes ADU-V19, ADU-S100, diABZI STING agonist-1 (Tautomerism), IACS-8779, IACS-8803, E7766, BMS-986301, GSK3745417, IMSA101, MK-1454 and SB 11285. ADU-V19 (RR–S2 cGAMP) is a newly-modified CDN. The novelty is human STING-targeted. As a derivative of STING ligand, ADU-V19 is free from phosphodiesterase by chemical modification and can target human STING. ADU-V19 share a similar function with those common agonists: producing type I IFN, maturating DCs and activating T cells [91]. ADU-S100 (ML RRS2 CDA or MIW815) is another STING agonist, which is similar to ADU-V19. ADU-S100 alone and co-injection of CPIs can both result in anti-tumor effects. The activation of CD8^+^ T cells and increased survival are observed respectively. What different from ADU-V19 is that ADU-S100 is undergoing phase I or phase II clinical trials (NCT03937141, NCT03172936 and NCT02675439). Tautomerism is a STING against with high selectivity. Subcutaneous injection of 2.5 mg/kg in vivo generates type-I interferon and pro-inflammatory cytokines. Tautomerism’s half-life is 1.4 h and its systemic concentration is higher than the half-maximal effective concentration (EC50) for mouse STING (200 ng/ml). Tautomerism exerts tumor growth inhibition and improves survival. Eight out of ten mice are witnessed tumor-free by the end of the study (day 43) [92]. IACS-8779 (C_21_H_25_N_9_O_10_P_2_S_2_) and IACS-8803 (C_20_H_23_FN_10_O_9_P_2_S_2_) are both STING agonists with robust systemic antitumor efficacy. Mice implanted bilaterally with B16 melanoma cells received intratumoral IACS-8779 injection unilaterally. Scientists observed that superior regression appears on the untreated tumor [93]. The following drugs (E7766, BMS-986301, GSK3745417, IMSA101, MK-1454 and SB 11285) are indicated in the National Cancer Institute (NCI) drug dictionary of National Institutes of Health (NIH) website (https://www.cancer.gov/publications/dictionaries/cancer-drug).

#### Clinical trials of cGAS-STING pathway agonists

cGAS-STING pathway agonists is a key activator, for the fact that hyperactivity of the pathway is significantly involved in tumor regression, prolonged survival time and enhanced immunity. Therefore, developing drugs targeting the cGAS-STING pathway is worth numerous dedication. Currently, important clinical trials of the cGAS-STING pathway agonists alone and a combination of CPIs are summarized in Table 1 [94–112].

**Table 1:** Summary of the cGAS-STING agonist clinical trials

Cancer tpye	Drug administration	Phase	NCT number	Allocation	Actual Enrollment	Status	Others	References
Adult Solid Tumor	ASA404 (i.v.)	I	NCT00003697	–	3 participants	Recruiting	–	[94]
Hormone Refractory Metastatic Prostate Cancer	ASA404	II	NCT00111618	Randomized	70 participants	Completed	Open Label	[95]
Non-small Cell Lung Cancer	ASA404 (i.v.) + Paclitaxel(i.v.) + Carboplatin(i.v.)	I	NCT00674102	–	15 participants	Completed	Open Label	[96]
Locally Advanced and Metastatic NSCLC	ASA404 (i.v.) + Paclitaxel(i.v.) + Carboplatin(i.v.)	I/II	NCT00832494	Randomized	105 participants	Completed	Open Label	[97]
Refractory Tumors	ASA404 (i.v.)	I	NCT00856336	Randomized	15 participants	Completed	Multicentre, Double blind	[98]
Solid Tumors	ASA404	I	NCT00863733	Non-Randomized	63 participants	Completed	Open-label, Single Group Assignment	[99]
Small Cell Lung Cancer	ASA404 (i.v.) + Paclitaxel(i.v.) + Carboplatin(i.v.)	II	NCT01057342	–	17 participants	Completed	Open Label	[100]
Advanced or Recurrent Solid Tumors	ASA404 (i.v.)	I	NCT01285453	–	9 participants	Completed	Single Group Assignment	[101]
Advanced/Metastatic Solid Tumors or Lymphomas	ADU-S100(i.t.)+/−ipilimumab(i.v.)	I	NCT02675439	Non-Randomized	47 participants	Active, not recruiting	Open Label, Multicenter Study	[102]
Advanced/metastatic solid tumors or lymphomas	MK-1454(i.t.)+/−pembrolizumab(i.v.)	I	NCT03010176	Non-Randomized	235 participants	Recruiting	Open-label, Multicenter Study	[103]
Advanced/Metastatic Solid Tumors and Lymphomas	ADU-S100(i.t.) + PDR001(i.v.)	Ib	NCT03172936	Non-Randomized	106 participants	Active, not recruiting	Open Label, Multicenter Study	[104]
Advanced Solid Tumors	GSK3745417 (i.v.) +/−pembrolizumab (i.v.)	I	NCT03843359	Non-Randomized	300 participants	Recruiting	Open-label	[105]
recurrent and Metastatic HNSCC	ADU-S100(i.t.) + pembrolizumab(i.v.)	II	NCT03937141	–	33 participants	Recruiting	Open Label	[106]
Advanced Solid Cancers	BMS-986301+/−(Nivolumab+Ipilimumab)	I	NCT03956680	–	75 participants	Recruiting	Open Label	[107]
Advanced Treatment-Refractory Malignancies	I: IMSA101(i.t.)+/−ICI; IIA: IMSA101 + IO therapy; IIA: IMSA101 + ICI	I/IIa	NCT04020185	Non-Randomized	115 participants	Recruiting	Open-label, dose escalation, dose expansion	[108]
Melanoma, HNSCC and Advanced Solid Tumor	SB 11285(i.v.) + Nivolumab	Ia/Ib	NCT04096638	Non-Randomized	110 participants	Recruiting	multicenter, open-label, dose-escalation, cohort expansion study	[109]
Non-muscle Invasive Bladder Cancer	E7766(i.v.)	I/Ib	NCT04109092	Non-Randomized	120 participants	Not yet recruiting	Open-label, Multicenter	[110]
Advanced Solid Tumors or Lymphomas	E7766(i.t.)	I/Ib	NCT04144140	Non-Randomized	120 participants	Not yet recruiting	Open-Label, Multicenter	[111]
Metastatic or unresectable, recurrent HNSCC	pembrolizumab(i.t.)+/−MK-1454(i.v.)	II	NCT04220866	Randomized	200 participants	Recruiting	–	[112]

Note: +/−, combination/alone

*Abbreviations*: *i.t.* intratumoral injection, *i.v.* intravenous injection

### Drug delivery system of STING agonists

Although there exist productive cGAS-STING agonists, they are still suffering their flaws decreasing biotherapeutic efficacy. The hydrophilicity, the vulnerability of enzymatic degradation and the negative charges all refrain from tremendous anti-tumor immunity. Therefore, developing an effective drug delivery system is an essence. The current cGAS-STING agonist delivery system has various forms, ranging from nanocarriers and microparticle to hydrogels. Generally, the smaller delivery vectors more easily transport drugs both locally and distantly. Oppositely, large vectors tend to function in situ after intra-tumoral administration. When choosing delivery vectors, there are two goals needed to be reached. First, at tissue levels, delivery vectors should stick around the cancer lesions to function. Second, at cell levels, drug delivery vectors penetrate the cell membrane arriving at the ER. Meanwhile, they must be protected from endosome decomposition. In the following part, we introduce three kinds of cGAS-STING agonist delivery system which have been widely investigated.

#### Nanocarriers

Although the activation of the cGAS-STING pathway fights against tumors, delivering agonists targeting tumors still confronts challenges. First, commercially available cGAS-STING agonists are produced soluble, which suffers from clearance easily. In contrast, the phospholipid bilayer of the cell membrane requires fusion substances to be liposoluble. Once soluble drugs are administrated, it is easier to be disseminated throughout the body and to be catabolized for clearance [113]. Even if scientists have tried several methods to overcome it, the results have limitations. A soluble agonist elicits the broadest cytokine response. Cytokine-related toxicity may lead to uncontrollable inflammation and mortal injury [114]. Another limitation is the non-achievement of the therapeutic effect of IT injection. Because non-injected tumor sites are also demonstrated to be regressed due to the activation of distant tumor-specific T cells [57]. Although intramuscular injection of cGAMP inhibits melanoma growth in mice [115], intramuscular injection is not a regular way of delivering drugs.

Next, CDNs are vulnerable to phosphodiesterases (PDEs) of which ecto-nucleotide pyrophosphatase I (ENPP1) has dominant cGAMP-hydrolyzing activity. Li [116] et al. found that ENPP1 locates on the basal lateral surface of the hepatocyte membrane and in the rough ER, instead of in the cytosol. As a Ca^2+^ store, ER provides an essence of Ca^2+^ for exerting hydrolytic action. Based on the evidence above, it is suggested that cytosolic CDNs should be transported across the ER membrane in a certain mechanism. Subsequently, scientists synthesized bisphosphothioate analog of 2′3′-cGAMP (2′3′-cG^s^A^s^MP) which is hydrolysis-resistant, only to find its increased biostability with higher activity after chemical modification. Apart from PDEs, CD39 and CD73 also contribute to the degradation of ATP. They catalyze ATP hydrolyzed by ENPP1 into AMP, which deepens the immunosuppressive microenvironment [117, 118]. In regards to cancer, ENPP1 overexpression causes different cancer phenotypes. For example, ENPP1-overexpressing breast cancer is involved in the tamoxifen resistance [119]. To address the vulnerability of CDNs, some nucleotide-based ENPP1 inhibitors and non-nucleotide-based ENPP1 inhibitors are developed. However, the high acidity of nucleotide-based ENPP1 inhibitors dampens its oral bioavailability. Off-target biological effects of those drugs would be magnified, because their structure resembles that of natural substrates [120]. These drawbacks of newly-developed ENPP1 inhibitors shed light on CDNs delivery systems using nanocarriers to improve drug efficacy.

Third, the negative charges of CDNs refrain CDNs from permeating through negatively charged cell membrane surface and hydrophobic membrane interior [121]. Since CDNs have more than one anionic phosphate group, such a characteristic limits STING agonists’ entry into the cytoplasm [122]. Cationic liposome with polyethylene glycol (PEG) was explored, because PEGylation enhances nanocarrier’s stability and persistence both in vivo and in vitro [122]. Hence, developing a nanocarrier-based drug delivery system is needed to allow CDNs as potential biotherapeutics penetrating through the cellular surface and arrive at the cytosolic region.

**Fig. 2:**
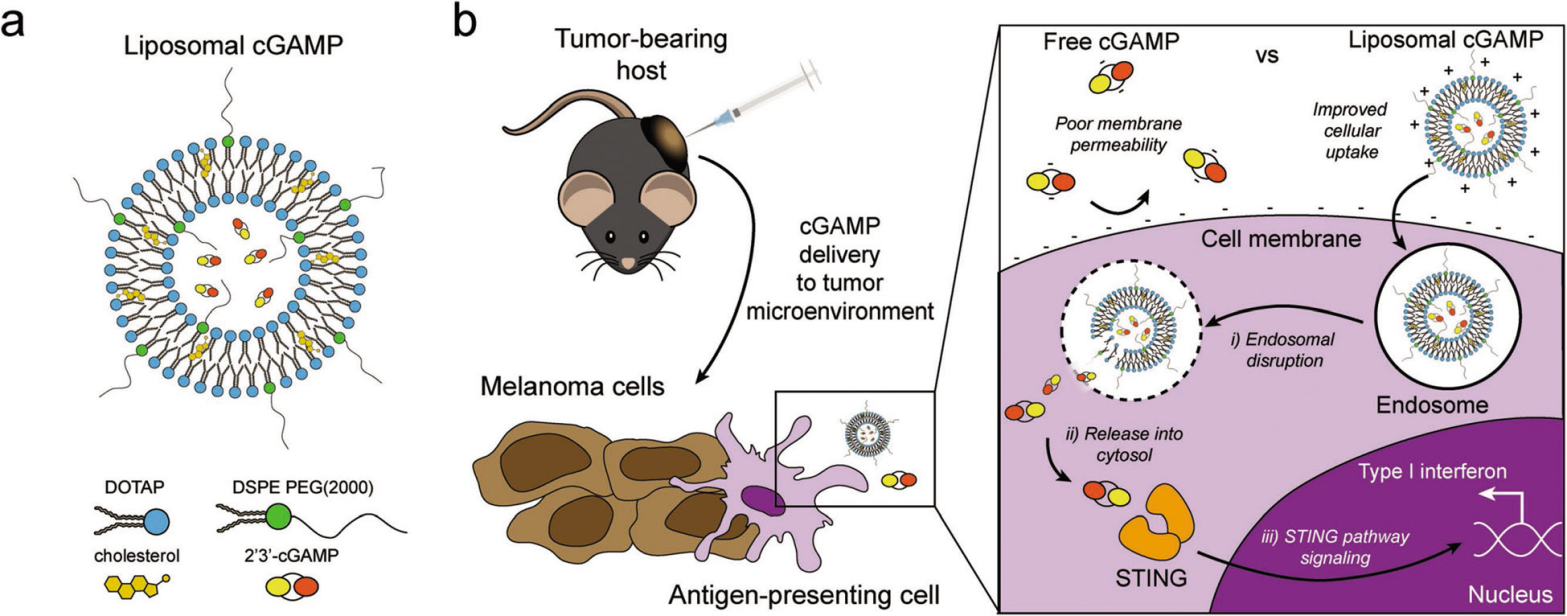
liposomal cGAMP structure and therapeutic strategy. a) DOTAP: 1,2-dioleoyl-3-trimethylammonium-propane, DSPE-PEG (2000): 1,2-distearoyl-sn-glycero-3-phosphoethanolamine-N-[methoxy (polyethylene glycol)-2000] polyethylene glycol; b) The injection of liposomal cGAMP in melanoma tumor-bearing mice are conducted. cGAMP alone cannot be transported into the cytosol. Liposomal cGAMPs are taken up, facilitating the release of cGAMP into the cytosol. Then, the cGAS-STING pathway is activated in the APC. Reprinted from [122], copyright (2017) Wiley

Liposomes is the first nano-biotherapeutics approved by FDA [123]. Liposomes-based delivery encapsulating STING agonists can activate immunologically active cells by cell localization. The function depends on the positively charged spherical vesicle structure exclusively for delivering nucleic acid. The injection of liposomal cGAMP in melanoma tumor-bearing mice are conducted. cGAMP alone cannot be transported into the cytosol due to the presence of two negative charges both on cGAMP surface and on the cell membrane surface. Liposomal cGAMPs are taken up, facilitating the release of cGAMP into the cytosol. Then, the cGAS-STING pathway is activated in the APC (shown as Fig. 2) [122]. On the one hand, liposomes function as vaccine adjuvants. For instance, the study investigated the amplified therapeutic effect of liposomal nanoparticles (NPs) encapsulating cGAMP, compared to that of soluble cGAMP alone. This study demonstrated that cGAMP-delivering NPs reinforce innate immune activation and anti-tumor effect. Particularly, in PD-L1-insensitive models of triple-negative breast cancer (TNBC), cGAMP-NPs induce a variety of inflammatory to fight against cancer, such as proinflammatory cytokines, nitric oxygen, type I IFN and so on. They are more or less involved in promoting M1-like polarization, MHC and costimulatory molecules expression, proliferative inhibition, apoptosis, migration regulation and antigen expression on cell surfaces [113]. Another case in point is that increasing dose of soluble CDNs causes systemic inflammatory toxicity. Hanson et al. found that the administration of CDNs reflects poor lymphatic uptake, because CDNs are eliminated out of tissues by the blood. Thus, scientists used PEGylated liposomal carriers to take place of soluble CDNs, only to find the activation of APCs and active T cell responses [124]. Jointly, a liposomal delivery system containing STING agonists can serve as vaccine adjuvants to enormously improve the anti-tumor effect and immune responses. On the other hand, nanocarriers can overcome the immunosuppressive TME. It is acknowledged that the inhalation of nanocarriers can fight against lung metastases by enhanced immunity. Hence, Liu et al. used liposome encapsulating cGAMP in mice models with lung metastases, indicating pro-inflammatory responses in those metastatic sites. Furthermore, this scientist made a comparison of such a delivery system accompanied by fractionated radiation and the delivery system alone. The results show the immunosuppressive TME exists both in tumors with or without radiation. Adding liposomes loaded cGAMP can control lung metastatic sites and prolong survival time of mice [88]. Therefore, in the immunosuppressive TME, liposomes with CDNs are effective to overcome the disadvantage of the immunosuppressive TME to generate anti-tumor immunity.

Polymeric nanocarriers are another promising nanocarriers to transport STING agonists for cancer biotherapy. They are simple and soft materials for drugs to be built into, because of convenient synthesis and surface structural modification. The feature determinates polymeric nanocarriers’ advantages: improved drug loading efficacy, good biodistribution and biodegradability, controlled pharmacokinetics and safe evaluation [125]. A supreme example is called polymersome. Polymersomes contain two important structures: an aqueous core and a vesicle membrane comprising amphiphilic di-block copolymer chains. The aqueous core is for loading hydrophilic drugs effectively. The vesicle membrane is pH-sensitive and membrane-destabilizing, which mediates the release of intracellular contents and endosomal escape of cGAMP. Polymersomes increase cGAMP activity in monocyte, macrophage and melanoma cell lines. Then, a T-cell- inflamed TME is elicited: increased tumor-infiltrating neutrophils (CD11b^+^Ly6c^+L^y6g^+^), the polarization from M2 macrophages to M1 macrophages (decreased CD206), overexpression of CD86 in tumor-drainaged lymph nodes, increased CD8^+^ and CD4^+^ T cells and increased CD8^+^/CD4^+^ T-cell ratio [126]. Another good example is a synthetic polymeric nanoparticle (NP), PC7A NP, developed by Gao et al. The PC7A NP is ultra-pH-sensitive, for the existence of tertiary amines with linear or cyclic side chains. PC7A NPs induce robust antigen-specific CTL, Th1 and Th2 responses. When taking a further look at the underlying mechanisms, Gao et al. identified that promoting antigen delivery and APC cross-presenting both by PC7A NPs jointly stimulate CD8^+^ T cell responses. In IFNα/βR^−/−^ mice, it is validated that PC7A NPs enhance immune responses by cGAS-STING pathway after APCs are activated in draining lymph nodes. Based on the above information about PC7A NPs, co-administration of a checkpoint inhibitor (anti-PD-1) in multiple tumor models (melanoma, colon cancer and human papilloma virus E6/E7) generates inhibitory effects of tumor growth and prolonged survival time [127].

#### Microparticles

**Fig. 3:**
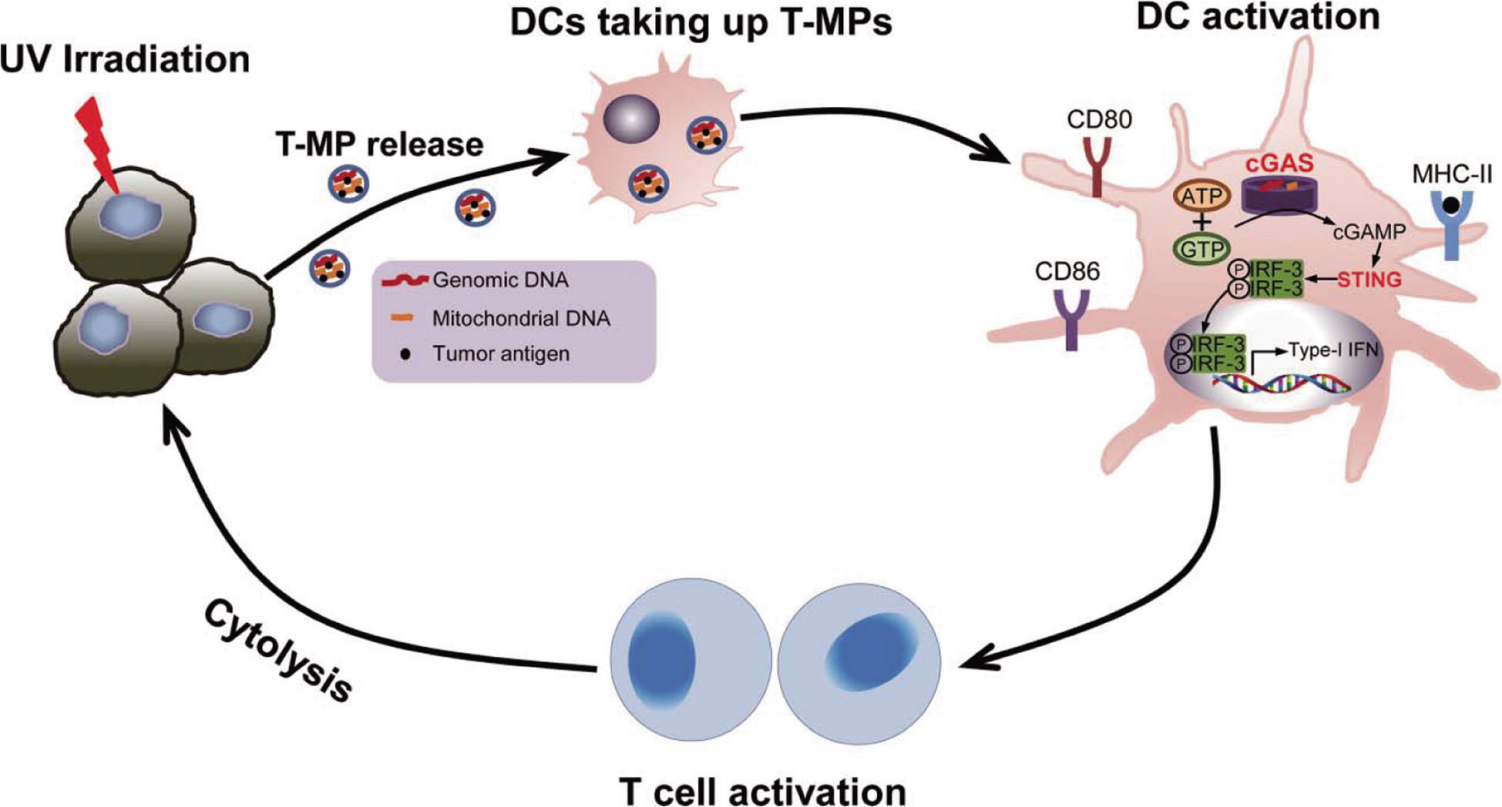
T-MPs function. Upon UV irradiation, tumor cells release MPs containing tumor antigens and DNA fragments. T-MPs present DNA fragments from cancer cells for APC, stimulating the production of type I IFNs by the activation of the cGAS-STING pathway. Type I IFNs, in turn, make DCs mature by up-regulating CD80, CD86 and MHCII. DCs present tumor antigens to T cells, eliciting anti-tumor effects. Reprinted from [129], copyright Taylor & Francis

The micromaterial-based STING agonist delivery system has been developed since 2015. In such a system, the microparticle (MP) is the most widely investigated in the cGAS-STING pathway. For example, microparticles generated from apoptotic cancer cells have been known as a delivery vesicle, carrying chemotherapeutic drugs to inhibit cancer cell growth without any side effect [128]. It is called tumor cell-derived microparticles (T-MP). T-MPs present DNA fragments from cancer cells for APC, stimulating the production of type I IFNs by the activation of the cGAS-STING pathway. Type I IFNs, in turn, make DCs mature by up-regulating CD80, CD86 and MHCII. DCs present tumor antigens to T cells, eliciting anti-tumor effects (shown as Fig. 3) [129, 130]. Besides, acid-sensitive acetylated dextran (Ace-DEX) polymeric MPs are another kind of MP delivery system. Compared to soluble cGAMP, Ace-DEX MPs encapsulating cGAMP increase 1000 times and 50 times the level of type I IFNs in vitro and in vivo respectively, leading to over 100-fold increase in antibody titers. Amazingly, the complex has no toxicity. By overcoming the localization of soluble cGAMP alone, Ace-DEX MPs encapsulating cGAMP potentiate the immunity and expand the germinal center [131]. In another study, Ace-DEX MPs encapsulate cGAMP, imiquimod, murabutide and poly (I:C). Among them, Ace-DEX MPs containing cGAMP are the most effective agent inhibiting tumor growth. The discovery exclusively indicates that NK cells are required for the anti-tumor effect both in TNBC and melanoma mice models [132].

#### Hydrogels

Hydrogels are locally-released anti-tumor drug carriers. The unique function is to bind and to retain water, thus forming viscoelastic gels in the aqueous solution [133]. It is reported that hydrogel scaffolds with cross-linking hyaluronic acid (HA) have biological advantages in releasing immunomodulatory drugs, such as biocompatibility and spatiotemporally-controlled drug release. Hydrogels loaded with 2′3′-cdi-AM (PS)2 (Rp,Rp) (“STING-RR”) injecting to intraoperative tumors have therapeutic benefits, however, it is not observed in unresectable tumors. The final result jointly supports that hydrogels loading immunomodulators affect postsurgical resection microenvironment by eliciting immune cells [134]. Linear polyethyleneimine (LPEI)/hyaluronic acid (HA) hydrogels loading cGAMP generate IFNβ and IL-6 in macrophage cells. Notably, the amount of IFNβ is 2.5 times higher than cationic liposomes. The corresponding IFNβ mRNA is elevated in mice spleens [135]. Matrigel is a thermo-responsive hydrogel comprising laminin and collagen IV. Matrigels have a series of advantages: soluble in water, absorption of soluble agents in water, the formation of a gel-like solid at body temperature and rapid degradation. Based on the feature, in the resection cavity, Matrigel incorporating STING ligands cure local tumors in the resection cavity of head and neck squamous cell carcinoma mice models. It is in the host STING expressing cells, not in cancer cells, that the production of type I IFNs contributes to the local control of residual lesions [136]. STINGel is a third peptide hydrogel for CDN intra-tumoral delivery. Multidomain peptides (MDPs) play a crucial role in creating hydrogels. MDPs generate anti-parallel β-sheets of peptide nanofibers in solution when β-sheets electrostatically crosslink with multivalent ions, thus expanding nanofiber networks. It is MDP that controls and extends CDN release. STINGel releases payloads more slowly than common collagen hydrogel does. The duration of releasing high concentration CDNs is simultaneously prolonged. A good thing is that released CDNs fail to recruit infiltrating cells, because immune-cell infiltration is cytotoxic. Meanwhile, cellular necrosis can be witnessed. In mice with oral cancer, the promotion of necrosis and the lack of cytotoxicity both enable cancer-bearing mice models tolerated. The immunotherapeutic mechanisms are the activation of cGAS-STING pathway and the release of danger-associated molecular patterns (DAMPs) from nuclear debris. DAMPs can recruit massive immune cells to the lesion [137].

## Conclusions

The cGAS-STING signaling pathway is characterized as the representative production of type I IFNs to elicit anti-tumor immunity. The activation of the cGAS-STING pathway transfer signals from the binding of ligand and receptor to the transcriptional level in the nucleus by phosphorylating second messengers, which renders STING a potential target for cancer biotherapy. The reason for it is that an effective STING- agonist delivery system could substantially enhance anti-tumor immunity. Besides, STING agonists are undergoing basic scientific researches and clinical trials. They have shown promising biological activity. Hence, to optimize the bio-therapeutic efficacy, delivering cGAS-STING pathway agonists to targeted tissues or cells is a method. Pathway agonists penetrate the cell membrane to arrive at Golgi for interaction. However, agonists commercially available have three disadvantages: easily to be cleared, easily to be enzymolyzed by PDEs and the similar charge property of agonists and organelle surfaces. Considering the disadvantages, developing a delivery system to overcome the difficulties and to target cells is imperative. Although studies about delivering agonists to tumor tissues and lymph nodes are investigated, agonists delivery systems are continuously developed for the improvement of cancer biotherapy. In the review, we summarize the cGAS-STING pathway agonists, including DMXAA that experienced failure in phase III clinical trials, CDNs that are widely accepted as common and effective cGAS-STING signal enhancers and other agonists that are investigated at an early stage. Also, we summarize the current drug delivery system, including nanocarriers, microparticles and hydrogels.

It is worth noting that the combination treatment of cGAS-STING agonists and CPIs can synergistically improve cancer biotherapeutic efficacy and decrease the risk of side effects. In mice, the agonist ADU-S100 co-administrated with CPIs is productive to magnify anti-tumor immunity. Moreover, most of the cGAS-STING agonist clinical trials are conducted with CPIs (pembrolizumab, ipilimumab and nivolumab) for cancer biotherapy. However, the underlying mechanism remains to be clarified. Now that cGAS-STING signal cascade transduction changes with the phosphorylation of multiple intracellular molecules after DNA sensing, mediating a series of biological process (inflammation, cell proliferation and apoptosis), clarifying mechanisms and optimizing combinational biotherapeutic strategies are both essential processes to solve drug safety problems for the maximization of drug efficacy. Furthermore, developing a more effective delivery system and designing rational cGAS-STING agonists are coordinatively important for improving cancer therapeutic effect.

In sum, the cGAS-STING pathway shows invaluable potentials in cancer biotherapy. Enhancing the immune ability to fight against cancer by delivering corresponding agonists with appropriate delivery systems can tremendously facilitate the combinational cancer biotherapeutic efficacy.

## Data Availability

Not applicable.

## Abbreviations

TREX1: Three prime repair exonuclease 1
RNase H2: Ribonuclease H2
cGAS: cyclic guanosine monophosphate-adenosine monophosphate synthase
dsDNA: double-stranded DNA
ATP: Adenosine 5´-triphosphate
GTP: Guanosine 5´-triphosphate
cGAMP: cyclic GMP-AMP
CDNs: Cyclic dinucleotides
ER: Endoplasmic reticulum
STING: Stimulator of interferon genes
IRF3: Interferon regulatory factor 3
IFN: Interferon
HSV-1: Herpes simplex virus-1
VSV: Vesicular stomatitis virus
PCV2: Porcine circovirus type 2
HIV-1: Immunodeficiency virus type 1
NTase: Nucleotidyl transferase
ERGIC: ER-Golgi intermediate compartment
TBK1: TANK Binding Kinase 1
MAP 3K14/NIK: Mitogen-activated protein kinase kinase kinase 14
IKK: IκB kinase
NFKB2/p100: Nuclear factor kappa B subunit 2
NFKBIA/IκB: NFKB inhibitor alpha
APCs: Antigen presenting cells
Th1: T helper cell
DCs: Dendritic cells
STIM1: Stromal interaction molecule 1
ISGs: Interferon-stimulated genes
MEFs: Murine embryonic fibroblasts
WT: Wild-type
SAVI: STING-associated vasculopathy with onset in infancy
S100: ADU-S100
CPIs: Checkpoint inhibitors
TAAs: Tumor-associated antigens
TME: Tumor environment
DMXAA: 5, 6-dimethylxanthenone-4-acetic acid
PARPi: Poly (ADP-ribose) polymerase inhibitors
TILs: Tumor infiltrating lymphocytes
NSCLC: Non-small cell lung cancer
OS: Overall survival
PFS: Progession-free survival
mAb: mono-antibody
DNL: Dock-and-Lock method
NPs: Nanoparticles
Tumor-VDAs: Tumor vascular disrupting agents
c-di-GMP: cyclic di-GMP
c-di-AMP: cyclic di-AMP
MDSC: Myeloid-derived suppressor cells
NCI: National Cancer Institute
NIH: National Institutes of Health
PDEs: Phosphodiesterases
ENPP1: Ecto-nucleotide pyrophosphatase I
PEG: Polyethylene glycol
TNBC: Triple-negative breast cancer
MP: Microparticle
T-MP: Tumor cell-derived microparticles
Ace-DEX: Acetalated dextran
LPEI: Linear polyethyleneimine
HA: Hyaluronic acid
MDPs: Multidomain peptides
DAMPs: Danger-associated molecular patterns
